# Primary epithelioid angiosarcoma of the jejunum presenting with severe anemia and distant metastasis: a case report and literature review

**DOI:** 10.3389/fonc.2025.1688462

**Published:** 2026-01-06

**Authors:** Shurong Liu, Junling Zhang, Xin Wang

**Affiliations:** Department of Gastrointestinal Surgery, Peking University First Hospital, Beijing, China

**Keywords:** anemia, angiosarcoma, biopsy, enteroscopy, jejunum

## Abstract

Angiosarcoma (AS), constituting approximately 2% of all soft tissue sarcomas, is an aggressive endothelial malignancy. We report on the case of a 72-year-old man admitted with persistent melena and severe anemia, ultimately diagnosed with jejunal angiosarcoma (JAS) and associated lung and bone metastases. Temporary stabilization of his hemoglobin level was achieved via fasting, hemostasis, and blood transfusion. The initial plain CT identified multifocal solid lung nodules, but failed to reveal a primary digestive system lesion. Subsequent small enteroscopy localized a submucosal bulge in the jejunum, 70 cm from the pylorus. Following partial small bowel resection, the pathological examination confirmed a 0.9-cm × 0.8-cm × 0.7-cm angiosarcoma invading the intestinal wall and lamina propria. Immunohistochemical (IHC) staining showed strong positivity for CD31(++), alongside CD34(vascular+) and ERG(+). This report reviews the recent literature on JAS, summarizing the diagnostic and therapeutic strategies.

## Introduction

Angiosarcoma (AS), a rare malignant soft tissue sarcoma (STS) originating from the vascular or lymphatic endothelium, manifests ubiquitously across anatomical sites, including the head and neck (27.0%), breast (19.7%), limbs (15.3%), trunk (9.5%), and the liver (6.0%). Subtypes are broadly classified based on their anatomical location: cutaneous, visceral, and deep soft tissue (1). Gastrointestinal involvement, predominantly in the jejunum and ileum, is uncommon (2). While jejunal angiosarcoma (JAS) is spontaneous in approximately 75% of cases, radiotherapy and chemical exposure are established etiologic factors (2–4). Diagnosis is challenging due to atypical early clinical symptoms (e.g., gastrointestinal bleeding, anemia, or abdominal pain) that are difficult to distinguish from those of other digestive tumors or ulcers. Pathologically, JAS lacks a typical cellular organization under light microscopy, and differentiation relies on immunohistochemical (IHC) staining (5). Its identifying surface markers include CD34, CD31, and ERG (6). The overall median survival time is 150 days, while the 5-year survival rate is only 20%–30%. The prognosis is extremely poor for patients with distant metastases (2, 7). However, JAS currently lacks clear treatment standards, and patients face a great survival challenge. This study reported on a case of primary JAS with lung and bone metastases. In addition, to provide a reference on the treatment of subsequently diagnosed patients, we reviewed cases of JAS in recent years and summarized the treatment concepts and therapeutic strategies.

## Case report

**Figure 1:**
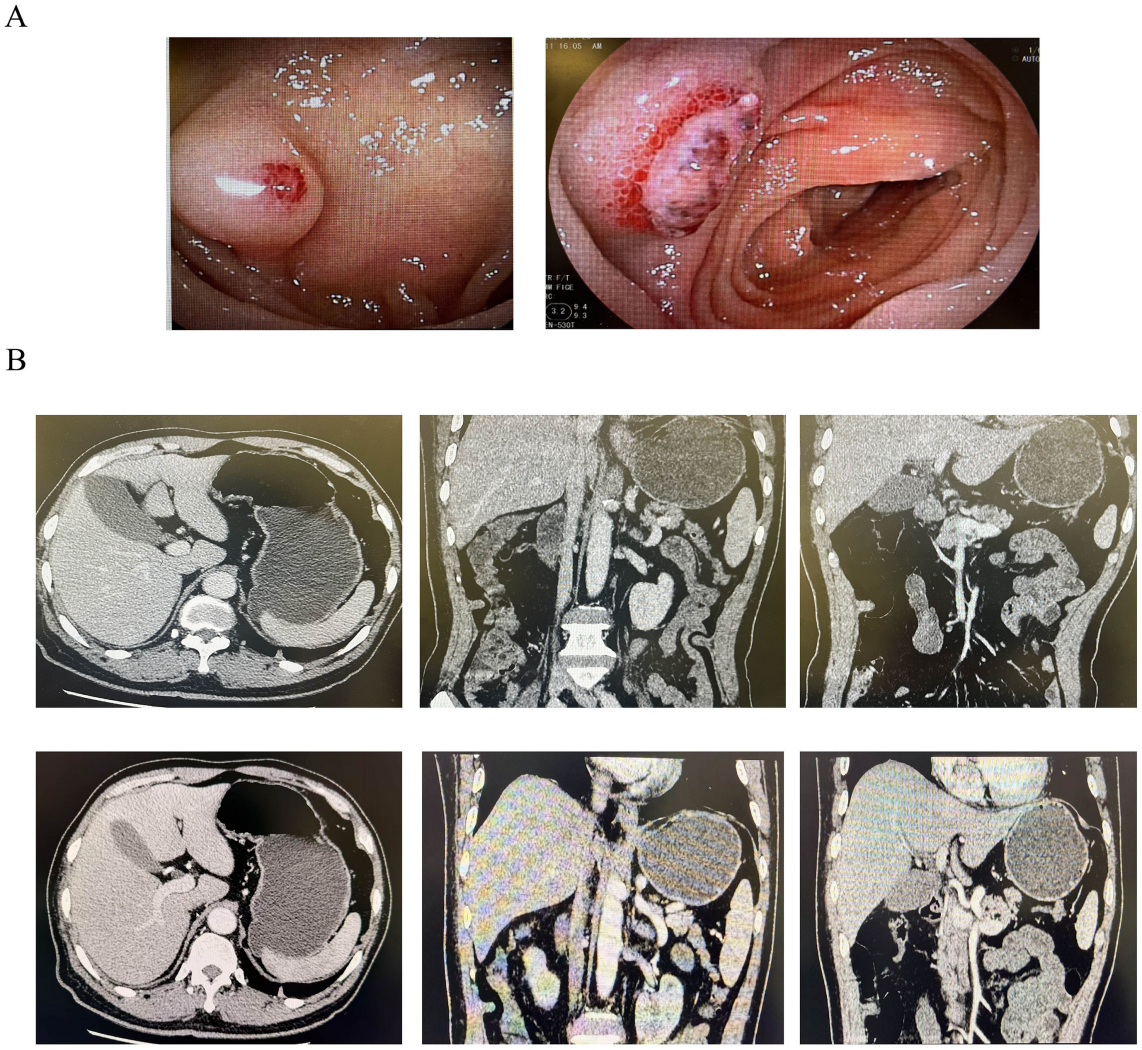
Contrast-enhanced CT and small enteroscopy before surgery. **(A)** Small enteroscopy showing a bulge with surface mucosal erosion and ulcerative changes, and visible blood vessels are exposed. **(B)** Plain (*up*) and contrast-enhanced (*down*) CT showing no hemorrhage or occupancy in the digestive system.

A 72-year-old man with hypertension presented to the Emergency Department with 1-month history of melena, following a 2-week course of antibiotics and ibuprofen prescribed for high fever and lung infection. The melena occurred one to two times daily and was accompanied by a persistent low-grade fever. The patient denied associated abdominal pain, diarrhea, nausea, or vomiting. Family history was negative for cancer. The patient denied any smoking or drinking habits. The initial laboratory assessment revealed severe anemia [hemoglobin (Hb) = 60 g/L], concurrent leukocytosis [white blood cell (WBC) = 10.36 × 10^9^/L), and a markedly elevated C-reactive protein (CRP = 64.21 mg/L). The patient’s tumor markers showed no significant abnormalities: alpha-fetoprotein (AFP) = 1.20 ng/ml, carcinoembryonic antigen (CEA) = 0.74 ng/ml, carbohydrate antigen 19-9 (CA 19-9) = 11.4 U/ml, carbohydrate antigen 72-4 (CA 72-4) = 1.00 U/ml, carbohydrate antigen 24-2 (CA 24-2) = 4.51 U/ml, total prostate-specific antigen (tPSA) = 0.518 ng/ml, free prostate-specific antigen (fPSA) = 0.089 ng/ml, and squamous cell carcinoma-associated antigen (SCCA) = 0.64 ng/ml. Following initial management—which included bowel rest, anti-infective and hemostatic therapies, and blood transfusion—the patient achieved temporary cessation of melena and symptomatic relief from anemia. Plain CT revealed a 4.8-cm soft tissue mass with calcified foci in the upper mediastinum, multiple small pulmonary nodules, and transverse colonic wall thickening. These findings suggested a primary gastrointestinal malignancy with distant metastasis. However, the abdominal enhanced CT was unremarkable for digestive tract abnormalities (**Figure 1B**), and subsequent gastroscopy and colonoscopy did not identify discernible bleeding sources in the stomach or colon. Oral small enteroscopy ultimately localized a submucosal bulge in the proximal jejunum, approximately 70 cm distal to the pylorus (**Figure 1A**). The bulge exhibited an eroded, ulcerated surface with exposed blood vessels, but no active hemorrhage. Consequently, biopsy was canceled due to the increased risk of bleeding.

**Figure 2:**
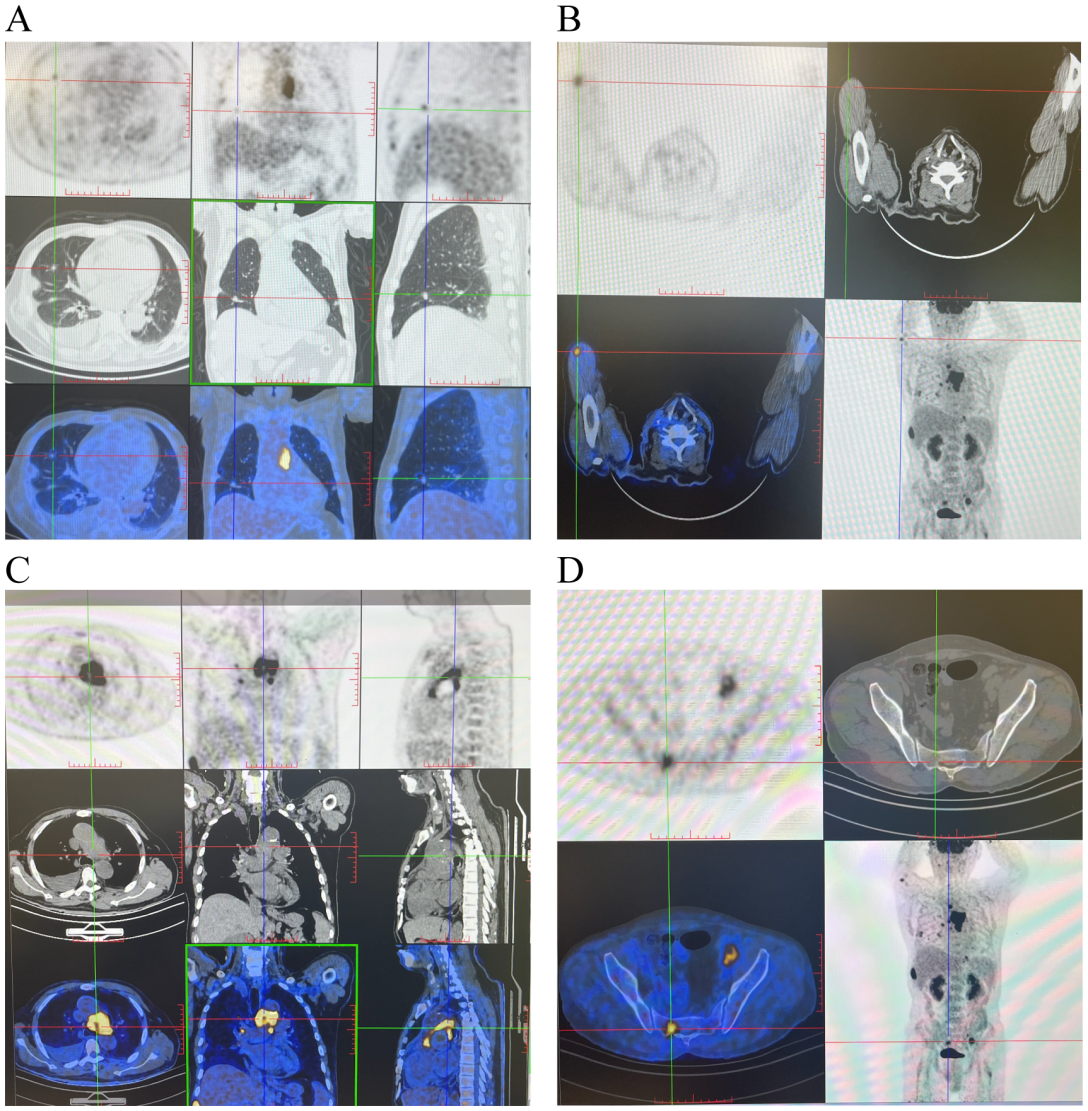
Specimens and immunohistochemical (IHC) results. **(A)** Jejunum specimen. *Yellow arrows* represent the two suspected bleeding points. **(B)** H&E staining of the resected jejunum, which is composed of highly atypical endothelial cells with numerous mitotic figures observed. **(C)** ERG expression in the tissue. **(D)** CD31 expression in the tissue. Both ERG and CD31 show significant positive expression in tumor tissues.

**Figure 3:**
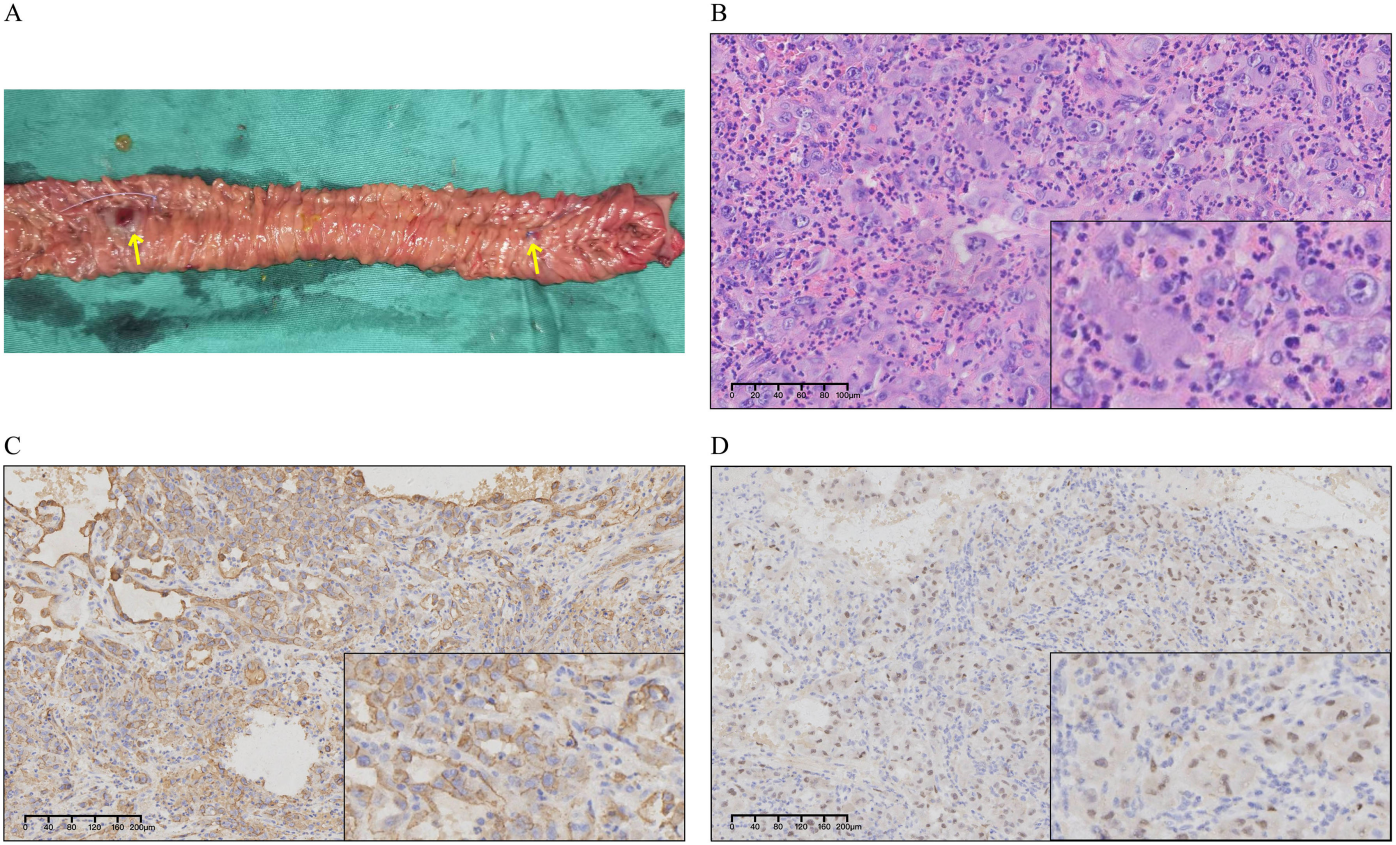
PET-CT after surgery suggesting multiple metastases. **(A)** One 5-mm nodule with high fluorodeoxyglucose (FDG) of multiple nodules in both lungs. **(B)** Muscles of the right upper arm with increased FDG. **(C)** Soft tissue density foci in the upper mediastinum with unevenly increased FDG. **(D)** Multiple foci of increased FDG in the sacrum with partial bone destruction. All of these lesion foci with elevated FDG were suspicious for tumor metastasis.

Intraoperative small enteroscopy identified two hemorrhagic lesions 50 and 65 cm distal to the ligament of Treitz in the proximal jejunum. Surgical management required excision of a 20-cm segment (**Figure 2A**) and subsequent side-to-side anastomosis. Histopathological analysis demonstrated a 0.9-cm × 0.8-cm × 0.7-cm tumor infiltration extending transmurally, from the lamina propria through the muscularis propria. The morphology of the tumor cells exhibited round, ovoid, and spindle shapes. IHC (**Figure 2B**) revealed the characteristic profile: CKPan(AE1/AE3)(+), vimentin(+++), LCA/CD45(−), CD34(vascular+), CD31(++), SMA(−), S-100(−), ERG(+), FLI-I(++), HHV8(−), DOG-1(−), CD117(−), p53(+), and Ki67(40%). The resulting diagnosis was an AS. Postoperative positron emission tomography (PET)-CT imaging strongly suggested widespread tumor dissemination, evidenced by multiple hypermetabolic lesions (**Figure 3**). The specific findings were as follows: bilateral pulmonary nodules, one measuring 5 mm, which displayed high fluorodeoxyglucose (FDG) avidity (**Figure 3A**); a hypermetabolic focus in the intermuscular space of the right upper arm (**Figure 3B**); upper mediastinal soft tissue foci exhibiting heterogeneous FDG uptake (**Figure 3C**); and multiple foci of increased FDG metabolism throughout the generalized skeletal system (**Figure 3D**), often associated with osteolytic destruction. These collective findings were highly suggestive of metastatic disease. The patient died 20 days after surgery due to gastrointestinal bleeding, hemorrhagic shock, and infection. Due to the patient’s poor physical condition and an unhealed surgical wound, he was not eligible for subsequent systemic treatment with radiotherapy or chemotherapy.

## Discussion

Due to its extremely low incidence, the literature on JAS primarily consists of case reports. Its nonspecific clinical presentation, including an unexplained gastrointestinal bleeding and abdominal pain (8, 9), often leads to misdiagnosis, as evidenced by one reported case initially identified as Crohn’s disease (10). JAS is usually difficult to detect with CT and gastroscopy. In this case, the final localization of the tumor was confirmed only after endoscopic examination of the oral small enteroscopy. The prognosis is exceptionally poor: the median survival is approximately 150 days, with the majority of patients succumbing within 1 year (11). Furthermore, JAS carries a significantly worse prognosis compared with AS at other primary sites (12). JAS is extremely insidious, given its aggressive nature and high malignancy, and the majority of patients present with lymph node or distant metastases at diagnosis, which also means a worse prognosis (13). Consequently, comprehensive preoperative systemic evaluation, such as PET-CT, and pathological confirmation are critical.

Differentiating JAS from entities such as poorly differentiated adenocarcinoma, gastrointestinal mesenchymal tumors, or melanoma is reliant on IHC. The markers for JAS include endothelial-related antigens such as CD31, CD34, ERG, CK, CK7, and factor VIII-related antigen (2). The differential diagnosis from melanoma is achieved by excluding melanocytic markers (i.e., S100, HMB-45, and Melan-A). In the current patient, the IHC results [CD34(vascular+), CD31(++), and ERG(+)] were consistent with these reports.

The management of JAS is guided by principles for advanced metastatic STS, emphasizing a multidisciplinary, individualized approach. Given this patient’s confirmed extensive distant metastases (lung, mediastinum, and bone), systemic treatment constitutes the primary therapeutic modality. Although R0 resection remains the only curative option for localized disease, the published data suggesting prognostic benefit in advanced disease lack statistical significance. In this metastatic case, surgery served two critical palliative and diagnostic functions: 1) to mitigate life-threatening symptoms such as continuous gastrointestinal hemorrhage and severe anemia resulting from tumor rupture or invasion and 2) to secure adequate tissue for definitive histopathological and molecular confirmation (epithelioid AS), thereby informing subsequent systemic strategies. Treatment focus must therefore prioritize systemic modalities. Standard systemic chemotherapy remains the foundation for the management of metastatic AS, demonstrably improving outcomes. The standard first-line regimen for metastatic STS is anthracycline-based, typically doxorubicin monotherapy or combined with ifosfamide (AI/ADI scheme) in patients with favorable performance status. Key second-line agents include taxanes (paclitaxel/docetaxel), which have demonstrated particular efficacy in cutaneous and head-and-neck variants, and the combination of gemcitabine and docetaxel. Due to the intrinsic vascular dependency of AS, anti-angiogenic targeted therapy is crucial. Multi-target tyrosine kinase inhibitors (MTKIs) such as pazopanib, a standard second- or third-line agent for metastatic STS acting via vascular endothelial growth factor receptor (VEGFR) and platelet-derived growth factor receptor (PDGFR) inhibition, are widely used. Other VEGFR inhibitors, including sorafenib and sunitinib, are employed clinically. Of note is that the rare epithelioid subtype in this patient shares morphological features with epithelioid sarcoma (14). Although not standard, this association warrants consideration of broader therapeutic avenues, such as epigenetic agents (e.g., EZH2 inhibitors including tazemetostat for *SMARCB1*-deficient tumors). Immunotherapy and molecular individualization immune checkpoint inhibitors (ICIs), specifically programmed cell death protein 1 (PD-1) blockade (e.g., pembrolizumab), have demonstrated response rates in certain AS cohorts, potentially linked to an elevated tumor mutation burden (TMB) or specific gene fusions (frequent in post-radiation and head-and-neck subtypes). Given the angiogenic dependency, combining ICIs with anti-angiogenic TKIs is a promising strategy to mitigate resistance and enhance efficacy. Future therapeutic strategies must prioritize molecular individualization: comprehensive genomic profiling is essential to detect actionable alterations such as *MYC* amplification (in cutaneous/radiation-induced AS) and *KDR* mutations/fusions and to assess programmed death-ligand 1 (PD-L1) expression and TMB, thereby guiding optimal targeted or immune treatment selection.

Future therapeutic strategies must prioritize individualized treatment. This requires genetic testing and molecular typing to identify patient-specific driver mutations and potential targets, thereby enabling the development of personalized regimens. The optimal approach involves integrating multimodal treatment combining surgery, radiotherapy, chemotherapy, targeted therapy, and immunotherapy to enhance patient survival and quality of life. Novel strategies, such as exploring the clinical application of epigenetic drugs (e.g., HDAC inhibitors) or investigating the preclinical potential of chimeric antigen receptor (CAR) T-cell therapy must also be pursued.

## Conclusion

This report details the case of a 72-year-old man presenting with JAS and widespread lung and bone metastases. His initial presentation involved cryptic gastrointestinal hemorrhage and severe anemia, ultimately necessitating surgical exploration and partial small bowel resection for definitive pathological diagnosis. Current therapeutic paradigms for AS are shifting rapidly, moving beyond traditional surgery and radiation to incorporate molecularly targeted agents and immunotherapy. Given the inherent rarity and high heterogeneity of JAS, optimizing management necessitates robust, large-scale clinical investigation and rigorous multidisciplinary collaboration. Precision medicine and novel therapeutic strategies are critical for improving patient outcomes.

## Data Availability

The raw data supporting the conclusions of this article will be made available by the authors, without undue reservation.
